# Comparative study of the effectiveness of EMDR G-TEP and CBT group protocols for the treatment of trauma in children exposed to a conflict context

**DOI:** 10.1192/j.eurpsy.2024.1378

**Published:** 2024-08-27

**Authors:** E. Dozio, C. Bizouerne, V. Wamba

**Affiliations:** ^1^ Action contre la Faim; ^2^Indipendent, Paris, France; ^3^Action contre la Faim, Bangui, Central African Republic

## Abstract

**Introduction:**

357 million children live in conflict zones. Children’s mental health is a major but complex issue as needs and interventions depend on the age of the child, caregivers, daily safety and protection, etc. EMDR and CBT are the recommended therapies to treat PTSD according to the WHO, but there is not enough standardized evidence-based protocol for children. Testing and evaluating trauma management systems for children is essential for trauma treatment interventions to be implemented in emergency contexts, such as war and conflict situations.

**Objectives:**

This research compares two intervention protocols for children aged 6 to 17 years suffering from Post Traumatic Stress Disorder after exposure to conflict traumatic events in the Central African Republic: the protocol “Kôno” developed by Action contre la Faim, based on a CBT and narrative approaches and the EMDR/G-TEP (Group Traumatic Events Protocol). After a psychoeducation session, the children were assigned to the ACF-KONO or EMDR/G-TEP groups for 5 sessions. The Child Psychosocial Distress Screener (CPDS) to measure general well-being and functioning and the Child Revised Impact of Events Scale (CRIES) to assess trauma, were administered before and after treatment.

**Methods:**

793 children participated in the research, 391 were included in the ACF-KONO protocol and 402 in the EMDR/G-TEP protocol. Both protocols have been shown to be equally effective in improving well-being and reducing traumatic symptoms. 185 children (90 ACF-KONO and 95 EMDR/G-TEP) were also re-evaluated after 5 months. The CPDS and CRIES-8 measurements reveal that the results are stable over time, with the use of both protocols. Detailed results will be presented.

**Results:**

This research contributes to the discussion on a framework for group protocols for children in mental health and psychosocial support interventions in humanitarian programs. The two protocols tested showed very good results in reducing symptoms of PTSD in children. How to choose between EMDR/G-TEP and CBT? What contextual and cultural adaptations are to be expected? Are there differences in children’s appreciation? And in that of mental health practitioners? Ideas for reflection will be shared.

**Conclusions:**

It is possible to widen access to therapeutic treatment of PTSD for children in emergency situations such as war and conflict. Further research in other contexts is needed. These studies should explore qualitative elements, such as children’s appreciations, but also the impact of these different protocols on the vicarious trauma of professionals involved in the treatment of children’s trauma.

**Disclosure of Interest:**

None Declared

